# Blunted cardiovascular reactivity may serve as an index of psychological task disengagement in the motivated performance situations

**DOI:** 10.1038/s41598-021-97670-0

**Published:** 2021-09-10

**Authors:** Maciej Behnke, Adrian Hase, Lukasz D. Kaczmarek, Paul Freeman

**Affiliations:** 1 Faculty of Psychology and Cognitive Science, Adam Mickiewicz University, 60-568 Poznan, Poland; 2 Department of Medicine, Université de Fribourg, 1700 Fribourg, Switzerland; 3 School of Sport, Rehabilitation and Exercise Sciences, University of Essex, Essex, CO4 3SQ UK

**Keywords:** Psychology, Human behaviour

## Abstract

Challenge and threat models predict that once individuals become engaged with performance, their evaluations and cardiovascular response determine further outcomes. Although the role of challenge and threat in predicting performance has been extensively tested, few studies have focused on task engagement. We aimed to investigate task engagement in performance at the psychological and physiological levels. We accounted for physiological task engagement by examining blunted cardiovascular reactivity, the third possible cardiovascular response to performance, in addition to the challenge/threat responses. We expected that low psychological task engagement would be related to blunted cardiovascular reactivity during the performance. Gamers (*N* = 241) completed five matches of the soccer video game FIFA 19. We recorded psychological task engagement, heart rate reactivity, and the difference between goals scored and conceded. Lower psychological task engagement was related to blunted heart rate reactivity during the performance. Furthermore, poorer performance in the previous game was related to increased task engagement in the subsequent match. The findings extend existing literature by providing initial evidence that blunted cardiovascular reactivity may serve as the index of low task engagement.

## Introduction

Competition is an integral part of life. Individuals compete to get a job, find a life partner, get into college, or win sports tournaments. Sometimes success requires only a single effort, but most often, success involves a complex and dynamic process involving performance and performance feedback at different time points. For instance, tennis players must win two or three sets consisting of six games to win the match and multiple matches to win a tournament. Thus, the competition comprises an ongoing process of winning and losing points, games, sets, and matches. A similar situation is observed in most esports, combat sports, or even team sports. Having experienced progress or obstacles, individuals must keep their motivation and maintain engagement at psychological and physiological levels to achieve ultimate success. However, sometimes individuals may disengage from the situation at hand and stop trying their best, which could harm their performance.

In this investigation, we examined task engagement in a multi-round performance at the psychological and physiological levels. Precisely, we tested whether reduced or absent task engagement at the psychological level (i.e., motivation to successfully engage the competitive situation) was associated with blunted cardiovascular reactivity to performance. This hypothesis was based on a recent review^1^ that integrated the phenomenon of blunted cardiovascular reactivity to stress^2^ with the predictions of the biopsychosocial model of challenge and threat^3^. Although the models conceptualize the blunted cardiovascular reactivity to stress as reduced or absent task engagement in motivated performance^2,3^, to date, no study has scrutinized these predictions in the sports domain. Furthermore, we explored how task engagement and performance outcomes influenced each other in the multi-round competition. To test our hypotheses, we focused on esports, which is the fastest-growing area in sports, and in which individuals compete with one another using video games.

### Task engagement and motivated performance

Challenge and threat psychophysiological models have become the leading frameworks for studying psychophysiological responses to performance^3–7^. A challenge state (measured at the cognitive and physiological levels) is beneficial to athletes, whereas a threat state is maladaptive^8,9^. However, before examining predictors and consequences of challenge and threat states, researchers should demonstrate that individuals were engaged in the task^3,4^. Task engagement reflects initial preparedness for action^3^. Individuals highly engaged in the task aim to invest considerable efforts, whereas individuals disengaged from the task plan to invest little or no effort in the task. Thus, task engagement can be considered the motivation to successfully engage with the upcoming task, which should result in an attentional and behavioral focus on the task^3^. In individuals who exhibit this task engagement, the context can be referred to as a motivated performance situation. Task engagement is usually observed on the physiological (cardiovascular) level. According to the challenge and threat models, task engagement involves increased sympathetic activation in the autonomous nervous system, indicated by increased heart rate (HR) and by shortened pre-ejection period (PEP) relative to a pre-task resting baseline^10^. A lack of such cardiovascular responses is interpreted as task disengagement^1^. For instance, studies have shown that participants who made fewer attempts to complete a subsequent impossible puzzle responded to the task with lower cardiovascular reactivity^11,12^.

Although task engagement is an essential part of challenge and threat models, it is also an understudied part of the models^9^. It is often limited to a simple *t*-test to indicate that at the group level, performers were engaged in the task and that the task did represent a motivated performance^13,14^. Not testing engagement at the individual level produces limitations to the challenge and threat literature because disengaged individuals who displayed blunted reactivity could be missed in group-level analyses. Only a few studies have excluded or accounted for participants who were not physiologically engaged in the task in their analyses^15–17^. Accounting for a third group of physiologically blunted individuals—that is, in addition to challenged (increased cardiac efficiency) and threatened (decreased cardiac efficiency) individuals—might improve understanding of psychophysiological reactivity to motivated performance situations.

Studies using a data-driven analytical approach have provided evidence that accounting for individuals without cardiovascular reactivity to motivated performance is justified. Precisely, studies have established that next to individuals who respond to motivated performance tasks with challenge or threat reactivity, there is a third group of individuals who display blunted responses; that is, no changes in cardiac function^18,19^. Blunted cardiovascular reactivity is operationalized as a lack of, or weaker than typical, cardiovascular response to a stressor^2^. Blunted cardiovascular reactivity to stress may reflect lack of effort or investment^20,21^ and has been linked to many adverse phenomena such as depression, anxiety, poor health, obesity, and cognitive ability ^22–26^, and poor sports performance^16^. In sum, blunted reactivity may reflect dysregulation of motivational systems within the brain^27^.

### Previous performance outcomes and subsequent performance

During motivated performance, there is a continuous feedback loop that determines task engagement, challenge-threat evaluations, physiological responses, and performance outcomes^3^. Based on past results, such as relative success or failure, individuals update their engagement and evaluations, which can impact subsequent performance.

The threat-provoked motivational disengagement hypothesis^1^ indicates that if an athlete performs poorly in a match and thinks that the next match will be an inevitable failure, then the athlete will lose goal relevance and consequently disengage from the task. If experienced chronically or severely, these negative consequences may result in stable motivational disengagement, that is, the general tendency to avoid similar future performance situations to prevent the aversive experience. However, if a motivated performance situation is unavoidable (e.g., in an ongoing tournament or match), then motivational disengagement may result in worse performance relative to motivationally engaged individuals^1^. A study of academy soccer players supported this idea, as athletes exhibiting the cardiovascular signs of motivational disengagement performed worse than engaged athletes, even those athletes who exhibited a threat state^16^.

A second possible scenario is that if an athlete loses or underperforms in a match, but remains optimistic about the next match, then the athlete will maintain or increase goal relevance and will engage in the task to bounce back from the perceived failure^28,29^. This may be due to optimism bias, in which in some individuals, optimistically biased judgments persist even after obtaining objectively insufficient results in the initial performance^30^. Even following failure, optimistic individuals persist in their motivations and confidence, and in turn, perform well in subsequent tasks^31^. Recently, two studies examined whether cognitive evaluations, physiological responses, and performance in a soccer penalty task^32^ and esports^13^ predicted cognitive evaluations and physiological responses in a second trial of the same task. In both studies, researchers did not find previous performance influenced subsequent evaluations or engagement. Evaluations and physiological engagement were relatively stable, such that evaluations and physiological engagement in one round were related to evaluations and physiological engagement in the next round and were not influenced by the previous performance^13,32^.

### Present study

The present study aimed to examine whether blunted cardiovascular reactivity serves as an index of psychological task disengagement. Furthermore, we explored the association between task engagement and performance outcomes in the multi-round performance in two ways: (1) how previous performance influences subsequent psychological task engagement and cardiovascular reactivity; and (2) how task engagement at both psychological and physiological level influences subsequent performance. Building upon theoretical models^1,3^, we formulated the following hypotheses. We expected that low motivation to engage with the performance (i.e., low psychological task engagement) would predict blunted cardiovascular reactivity (i.e., the lack of physiological task engagement as indicated by no change or a decrease in HR). Next, we held competing hypotheses for the motivating role of previous game results. First, we tested the threat-provoked disengagement hypothesis, which indicates that initial poor performance would produce task disengagement measurable at cognitive and cardiovascular levels^1^. This prediction was based on the theory of challenge and threat states in athletes, which suggests that task disengagement may result from threatening experiences in motivated performance situations such as poor performance^5^. On the other hand, it is possible that following failure on a given task, task engagement and performance would remain unchanged or would improve bouncing back from failure, as long as individuals believed in future success^28,29^. This prediction was supported by the observation that people in sports contexts are optimistically biased about future success^30^. Finally, based on a recent review, we expected that reduced or absent task engagement—on both motivational and cardiovascular levels—would result in worse performance relative to engaged individuals^1^. Although the biopsychosocial model of challenge and threat proposes that HR reactivity is not related to performance outcomes^3^, we expected that both challenged and threatened participants would outperform physiologically disengaged participants, in line with Dixon and colleagues’ recent study^16^.

The uniqueness of our approach stems from accounting for both motivational and cardiovascular indexes of task engagement. Unlike challenge and threat states, which have been measured as self-reports and cardiovascular responses^9^, task engagement has not been studied with both measures (except for^33,34^, who measured self-reported task importance). In our study, we focused on esports performance, where participants played five matches in the video game FIFA 2019 (a soccer game; Electronic Arts Inc. 2019). Playing esports is an example of a motivated performance situation characterized by active coping with situational demands under evaluative pressure in pursuit of a self-relevant goal^3^, and has been previously examined in the psychophysiological stress literature^35^.

## Methods

The data for this study were derived from a project that examined the effects of emotions on the challenge and threat evaluations, physiological responses, and performance outcomes. The part of the project relating emotions to challenge and threat variables (i.e., cardiac output reactivity) and esports performance has been presented in another article^36^. We demonstrated that the emotions had no effects on motivation and cardiovascular reactivity and that challenge/threat evaluations, cardiac output reactivity, and approach motivation were related to better performance^36^. In this manuscript, we aimed to test novel hypotheses related to psychophysiological task engagement—the understudied part of the challenge/threat framework. The results concerned with cardiovascular indexes of challenge and threat were reported elsewhere^36^.

### Participants

Participants were 241 male gamers between 18 and 37 years old (*M* = 23.63, *SD* = 3.63). A power analysis indicated that to detect the expected small effect sizes *B* = 0.10, with a power of 0.80, at least 200 participants would be required in Structural Equation Models analyses^37^. Gamers reported that the time they spent playing the game during a regular week ranged from 1 to 34 h per week (*M* = 7.24, *SD* = 5.78). Each participant provided written informed consent and received two vouchers for a cinema ticket for participation in the study. The gamers had no known history of cardiovascular or respiratory disease and had normal or corrected vision. The body mass index (BMI) of the gamers ranged from 16.85 to 35.55 kg/m^2^ (*M* = 24.55, *SD* = 3.19). We asked participants to change the date of the laboratory visit if they suffered from illness or had undergone a major negative life event and to abstain from strenuous exercise, eating, and caffeine for two hours before the laboratory visit. We made the above restrictions to limit factors that could affect cardiovascular function or esports performance. More details about participants are presented in^36^. The study was approved by and performed in accordance with guidelines and regulations of the Institutional Ethics Committee at Faculty of Psychology and Cognitive Science, Adam Mickiewicz University.

### Procedure

**Figure 1:**
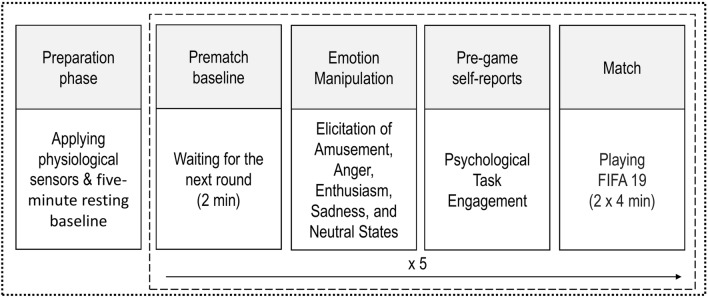
Study procedure. The dotted frame represents the study procedure. The dashed frame represents the repeated procedure of each of five rounds. The impact of 2-min emotion elicitation on the performance was not relevant to the present study aims and is reported in another article^36^.

Gamers played FIFA 19 individually in a soundproof and air-conditioned room. After arriving at the laboratory, the participants gave their informed consent, and the researcher attached the sensors to obtain cardiovascular measurements. We presented all instructions and collected answers using a computer with a 23-inch screen. The experiment began with a five-minute resting baseline, during which we asked the players to sit still. Next, participants completed five rounds consisting of (1) two minutes of pre-match baseline; (2) emotion elicitation with film clips; (3) reporting the motivation to engage in the performance (and other psychological variables not relevant to the present aims); (4) playing the video game (Fig. 1). The impact of the 2-min emotion elicitation on the performance was not relevant to the present study aims and was reported elsewhere^36^. As in traditional soccer, the team that scores the most goals wins the match in FIFA 19. All participants selected and controlled their favorite team against Real Madrid (controlled by computer) in a Classic Match mode at difficulty level “professional” to standardize conditions. Each match lasted eight minutes (two 4-min halves). After the fifth round, the experimenter removed the biosensors and debriefed the participants.

### Measures

#### Psychological task engagement

We operationalized psychological task engagement as the self-reported preparedness to engage with the upcoming task^3^. Participants reported their pre-game motivation to engage with the task with the 5-item *Effort/Importance* subscale from the Intrinsic Motivation Inventory^38^. The Intrinsic Motivation Inventory scale includes items such as “I will put a lot of effort into the next match” or “It is important to me to do well at this match”. Participants responded on a 7-point scale ranging from 1 (*strongly disagree*) to 7 (*strongly agree*). The data showed good internal consistency (Cronbach’s α = 0.88).

#### Blunted cardiovascular reactivity

Reactivity was assessed as the difference between the mean during the baseline (pre-stress) period and the mean during the stress period^39^. For instance, heart rate reactivity was the increase in heart rate from baseline to a stressful task. Blunted cardiovascular reactivity refers to a lack of, or weaker than typical, cardiovascular reactivity to the period of interest, which might be a stressor^2^. Cardiovascular biosignals were recorded continuously and noninvasively with electrocardiography with a sampling rate of 1000 Hz (Vrije Universiteit Ambulatory Monitoring System; VU-AMS, the Netherlands). We placed pre-gelled AgCl electrodes (Kendall Abro, H98SG) in standard configuration^40^. We processed the raw data with VU-AMS Data, Analysis & Management Software 3.0 (VU-DAMS 3.0). R peaks were identified automatically by the VU-DAMS 3.0 software, flagging skipped or spurious beats. Next, we visually checked and adjusted all R-peak markers to exclude outliers due to artifacts or ectopic myocardial activity, when necessary. Based on the intervals between R peaks, VU-DAMS 3.0 software calculated the heart rate (HR). HR was scored using 120-s ensemble averages (pregame baselines and matches).

To test whether gamers displayed blunted reactivity, we compared the HR levels from prematch baselines and matches. In our study, we dichotomized the reactivity to each match as blunted or not blunted reactivity rather than using reactivity as a continuous variable. Our approach is in line with the biopsychosocial model of challenge and threat, which proposes that physiological task engagement indicated by a significant HR increase (i.e., non-blunted reactivity) is necessary to assess the cardiovascular challenge and threat responses^3^. To test this, we calculated Cohen’s *d* for pairwise comparisons for a single match (effect size for the difference of means and standard deviations of HR level between pre-match baseline and match) for each gamer, resulting in 1205 possible pairwise comparisons. We reported the results of the pairwise comparisons as effect sizes (Cohen’s *d*) with confidence intervals (95% CI). We dummy coded the blunted cardiovascular reactivity variable, namely if the 95% CI for the effect sizes of HR reactivity included zero and/or the difference was significantly negative, then blunted cardiovascular reactivity was equal to 1; and if the 95% CI for the effect sizes did not include zero and the difference was positive, then blunted cardiovascular reactivity was equal to 0. After excluding participants with missing data, we found that in 57% of matches, participants displayed blunted cardiovascular reactivity. The mean HR reactivity among gamers who displayed blunted cardiovascular reactivity to the match was *M* = − 2.41, *SD* = 2.87, whereas among gamers who did not display blunted cardiovascular reactivity to the match was *M* = 4.10, *SD* = 3.57.

#### Performance

The performance level was operationalized as the number of goals scored minus the number of goals conceded during the match, with a higher score indicating better performance.

### Analytical strategy

#### Manipulation check

First, to test whether the gaming task was a motivated performance situation, we used repeated-measures analysis of variance (rmANOVA) with Greenhouse–Geisser correction and calculated effect sizes (*η*^*2*^). We compared the HR level during the match to HR baseline levels before the match. Increased HR level during the match, when compared to the pre-match baseline, indicated that the testing situation did represent motivated performance.

#### Primary analysis

To examine the hypotheses, we used structural equation modeling with maximum likelihood estimation with robust standard errors (MLR) using mPlus 8.1^41^. We regressed the current performance level on the mediators (psychological task engagement and blunted cardiovascular reactivity) and performance from the previous round. Furthermore, we controlled for psychological task engagement from previous match while testing the association between blunted cardiovascular reactivity and psychological task engagement. To account for the non-independence of observations, we nested four rounds of responses within individuals^42,43^. For psychological task engagement, we created a latent variable with five items. We calculated RMSEA and CFI, the recommended fit indexes for the MLR estimator. RMSEA values of < 0.08, along with CFI values of > 0.90, indicate acceptable fit^44^. After eliminating participants with missing data, we analyzed 896 rounds of responses (previous match results, psychological task engagement, cardiovascular reactivity, and the match results at a given time) nested within 228 gamers as each participant played five matches.

## Results

### Manipulation check

We found that gaming reflected a motivated performance situation characterized by increased HR (*M* = 76.23, *SD* = 11.42) when compared to pre-film baseline (*M* = 75.68, *SD* = 10.49), *F* (1, 1148) = 15.571, *p* < 0.001, η_p_^2^ = 0.013. Table 1 present the means and standard deviations for the average HR levels of pre-match baselines and match values.

**Table 1:** Means and standard deviations for HR levels for pre-match baselines and matches.

Round	Pre-match baseline		Match	
	*M*	*SD*	*M*	*SD*
1	76.92	11.30	79.15	11.95
2	76.10	10.64	77.52	11.52
3	75.87	10.37	76.03	11.23
4	75.34	10.20	74.57	10.93
5	74.62	10.30	73.85	10.71

### Primary analysis

**Figure 2:**
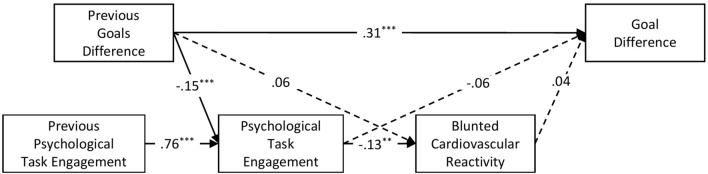
Path Model for Role of Task Engagement in Motivated Performance*. Note.* Blunted cardiovascular reactivity coded 0 = not blunted, 1 = blunted. Dashed lines represent insignificant paths. *p < 0.05, **p < 0.01, ***p < 0.001.

The structural equation model fitted the data well, RMSEA = 0.07, 90*%* CI [0.06, 0.08], CFI = 0.95 (Fig. 2). Descriptive statistics and correlations are presented in Table 2. Gamers who reported lower psychological task engagement in the gaming performance were more likely to display blunted cardiovascular reactivity during the match, β = − 0.13, 95% CI [− 0.21, − 0.05]. Furthermore, poor performance in the previous match resulted in increased psychological task engagement with the next performance, β = − 0.15, 95% CI [− 0.20, − 0.09]. We also found an indirect effect of previous performance outcome on blunted cardiovascular reactivity, namely gamers who performed poorly in the previous match were less likely to display blunted cardiovascular reactivity in the next match indirectly via higher psychological task engagement, β = 0.02, 95% CI [0.01, 0.03]. Finally, gamers that played well in the first match were more likely to perform well in the next match, β = 0.31, 95% CI [0.25, 0.38]. However, neither psychological task engagement, β = − 0.06, 95% CI [− 0.13, 0.01], nor blunted cardiovascular reactivity β = 0.04, 95% CI [− 0.03, 0.10] were related to subsequent performance outcomes. Furthermore, we reran the analysis controlling for the emotion manipulation and found no significant change from the reported results.

**Table 2:** Descriptive statistics and correlations among study variables.

	*M*	*SD*	1	2	3	4
1. Previous psychological task engagement	0.74	1.60	–			
2. Previous goal difference	28.50	5.29	− 0.07*	–		
3. Psychological task engagement	28.55	5.69	0.74***	− 0.18**	–	
4. Blunted cardiovascular reactivity	0.62	0.49	− 0.11**	0.08*	− 0.11**	–
5. Goal difference	0.77	1.62	− 0.10**	0.33**	− 0.09**	0.07*

Mean for blunted cardiovascular reactivity multiplied by 100 indicates the percentage of observations with blunted cardiovascular reactivity. *p < 0.05, **p < 0.01, ***p < 0.001.

## Discussion

In this investigation, we examined the predictions of the latest review regarding the relationship between task engagement and blunted cardiovascular reactivity^1^. We tested the hypothesis that reduced motivation to engage with the performance situation manifests itself as blunted cardiovascular reactivity to stress and may predict and be predicted by poor performance. Our main findings include:

1. Motivational (subjective) and cardiovascular indexes of task engagement were related;
2. Self-reported motivation and blunted cardiovascular reactivity did not predict subsequent performance outcomes;
3. Poor initial performance led to increased motivation to engage with the next performance and, in turn, to rarer blunted cardiovascular reactivity during the next performance;
4. A new method for calculating blunted cardiovascular reactivity that could be used to differentiate between motivated participants in challenge/threat studies and those who do not meet this theory-based motivational criterion for challenge/threat response to occur.

Our findings support the prediction of the biopsychosocial model of challenge and threat that psychological task engagement is related to cardiovascular task engagement response^1,3^. Previous studies indicated that blunted HR reactivity was related to a lack of behavioral perseverance^11,12,45^, but not self-reported perseverance^11^. We found that blunted HR reactivity may indeed serve as an index of psychological task disengagement in motivated performance contexts. Although it is widely used as a supporting assumption in challenge and threat research (by allowing researchers to test task engagement via the analysis of HR and ventricular contractility), this prediction has not been extensively tested before.

We found that individuals who performed better in the previous match displayed lower motivation and, consequently, a greater chance of blunted cardiovascular reactivity during gaming performance. These findings support the hypothesis that individuals were motivated to bounce back from losing the match^28,29^. Our findings may indicate that gamers were not discouraged by losses, but they maintained motivation to perform well in subsequent tasks^31^. Furthermore, our results may suggest that gamers followed the conservation principle when they were investing their effort^19^. According to motivational intensity theory^19^, individuals are motivated to avoid wasting their energy and are willing to invest only as much energy as required for successful task execution. Due to the nature of football, such behavior could quickly lead to a critical event such as conceding the losing goal, which would provoke greater investments of effort in the next game. On the other hand, having attained one’s goal in the previous game could explain the phenomenon of task disengagement in the next. Precisely because the next game might no longer be as self-relevant after a victory, task engagement would therefore not ensue.

We did not find support for the threat-provoked motivational disengagement hypothesis. Although we regarded poor performance as a threatening event, it could also be that losing the previous match did not constitute enough of a threat to elicit the threat-related motivational disengagement. Hase and colleagues^1^ predicted that repeated or severe experiences of threat states—such as series of losses or a loss to a seemingly undefeatable opponent—might be more likely to trigger motivational disengagement than single or light instances of threat states. Although there is evidence to suggest that extremely difficult tasks do indeed provoke blunted cardiovascular reactivity^46^, no previous empirical research has examined the effects of repeated threat states on motivational engagement. Our findings would be more robust if we used consecutive trials separated by longer recovery intervals. Moreover, as blunted cardiovascular reactivity has been previously linked to clinical depression^22,25^, it would be interesting to see whether depressed individuals reach a “motivational breaking point” earlier. Precisely, whether they experience a disengagement-provoking threat state earlier or whether less threatening experiences are required to provoke disengagement from a task.

Our findings did not support the prediction that blunted cardiovascular reactivity due to reduced task engagement explains poor subsequent performance^16^. This poses the question for future studies of whether one should distinguish between adaptive and maladaptive forms of task disengagement. Unlike previous studies that indicated maladaptive disengagement in sports^47^, we found a neutral disengagement pathway that did not harm the performance outcomes. As indicated in previous studies, individuals evaluated the upcoming task and performed at a relatively stable level, with a limited influence of previous performance^13,32^. On the other hand, the present study provided insights into an adaptive re-engagement pathway after a poor performance. Increasing task engagement after poor performance might be a homeostatic response in healthy individuals who noticed that they deviated from their average performance level. This is consistent with motivational intensity theory^48^, which predicts that motivated performance underlies a conservation principle, where the motivational arousal or mobilization of energy is only as strong as necessary to produce the desired outcome. Thus, a defeat might only do as little as alerting individuals that motivational arousal needs to be increased for the next match, as long as these individuals are not struggling with threat-related motivational disengagement. In summary, we found that gamers were motivated to bounce back after failure.

Our study also has an important methodological implication. We dichotomized the reactivity to each match as blunted or non-blunted reactivity, rather than using the reactivity as a continuous variable, which is the most common approach in the literature^49^. We used binary operationalization of blunted reactivity because, in this way, it could be used to exclude disengaged participants in challenge/threat studies. One of our goals was to validate the analytical strategy used by some authors, which involves excluding participants who displayed blunted reactivity to the performance (i.e., disengaged individuals) from further analysis^15–17^. By showing the relationship between blunted cardiovascular reactivity and psychological task disengagement, the current findings provide support for this approach.

Furthermore, gamers in our study displayed blunted cardiovascular reactivity to performance in a relatively large number of matches (57%). In line with previous studies, this highlights the necessity to account for blunted physiological responses in motivated performance situations rather than a sole focus on relative challenge-threat^15–18^. We encourage future studies to account for the blunted group and to refrain from performing a simple *t*-test to indicate that performers were engaged in the task at the group level only^13,14^. Previous studies have done this by excluding the blunted group from the analysis and focusing only on challenged and threatened participants^15,17^ or by including the third group in the analysis^16,19^. Since the blunted group might be a considerable proportion of all participants, they should not be ignored.

### Limitations and future directions

The study has several limitations that bear noting. First, to test our hypotheses, we ran a secondary analysis of existing data^36^. We reanalyzed archival data (collected from May to July 2019) to test a new theoretical proposition published after the original data collection^1^ (published online on April 24, 2020). After reading the review article, the first author (MB) contacted out the first author of the review article^1^ to test the new theoretical perspective with archival data. Furthermore, we changed the strategy for inferring participants' engagement from the group-level perspective presented in our previous study^36^ to the individual-level perspective presented in the present study. This was motivated by the recent proposal for a more detailed analysis of task engagement than is usually done in the challenge/threat literature^1^. Using high-quality pre-existing data (e.g., databases) to ask novel questions is an increasingly accepted strategy to advance science due to its feasibility, low costs, and low burden on the sampled population^50–52^.

Second, we limited our focus to HR as a specific measure of physiological task engagement, and we did not include PEP as a second index. This is because the apparatus used in this study did not provide standard deviations for PEP in each examined period, and thus we were unable to calculate the PEP pairwise comparisons for each match as we did with HR. However, previous studies have shown that HR and PEP are equally predictive for task engagement^34,53^. In future studies, it may be helpful to use HR, PEP, and self-reported measures of task engagement to obtain a broader picture of task engagement.

Third, although we found that gamers displayed higher HR levels during the matches than during the pre-match baselines, the effects found should be interpreted as small when interpreted on a group level. Thus, the statistical significance of the effect may not translate to the large practical significance of the observed effects. In future studies, researchers could validate our approach to infer its practical utility. .

Fourth, we focused on a single esports context. Although esports resemble traditional sports in terms of psychological challenges, they differ significantly in terms of physiological challenges. Gaming takes place in a seated position, which requires relatively less energy than running and controlling a ball. In this respect, esports is more like precision sports such as snooker, darts, or curling. However, in terms of cognitive and psychological requirements, a soccer video game involves creative play and quick decision-making, as does its traditional counterpart. Esports gaming represents a competitive motivated performance situation. It differs from traditional stressors used in cardiovascular reactivity research (e.g., Trier Social Stress Test^54^). The typical stress task used in the laboratory aims to maximize ecological validity by mimicking real-life stressful situations, e.g., by including social evaluation and provoking high cognitive effort. Although esports competitions include high social evaluation and required cognitive effort, the present study might have lacked ecological validity due to being less socially evaluative and cognitively pressurizing than a real esports competition. Future research can improve understanding of the relationship between engagement and performance by replicating our findings in the context of other sports.

Fifth, we did not include a separate recovery period after each match. The gaming situation could have impacted the next pre-match baseline level and, in turn, the calculation of the blunted reactivity for individual matches. For example, good and poor performance, as well as associated positive and negative emotions, could have had an impact on HR levels during the subsequent baseline^55^. Future studies could address this issue by introducing a separate recovery period after each match that would ascertain that the criteria for recovery were met. Moreover, it might be also feasible to extend the duration of the prematch baseline and treat the first half of the break between matches as a post-match recovery period and the second part of the break as a prematch baseline period.

Sixth, we measured the cardiovascular responses during the performance^13,56,57^ rather than before the performance^14,33^. Both approaches have their specific advantages and limitations that should be considered in assessing the fit of the approach with the current research aims. We agree with other authors who advocated the measurement of cardiovascular reactivity during performance because this is the actual period where blunted reactivity efficiency is expected to influence performance outcomes^36,58^. Future studies could compare whether pre-performance indexes of task engagement are more predictive of performance outcomes than performance-based indexes.

Seventh, we did not account for individual differences in our analysis. Although we speculated optimistic beliefs as one of the characteristics that could moderate the effects of poor performance on subsequent task engagement, we did not measure them in our study. Future studies might compare whether individuals with more optimistic beliefs are more resistant to the adverse effects of losing initial matches. Furthermore, studies might account for depressive symptoms linked to blunted reactivity^22,25^. Finally, all gamers in our study were young adult men. Although consistent with the typical environments of sport-type esports (where up to 98% of gamers are male^59^), future studies could focus on whether the results generalize to younger and female gamers.

## Conclusion

In summary, this study provides initial evidence for the link between psychological and cardiovascular task engagement as postulated by the biopsychosocial model of challenge and threat and motivated performance. Strengths of this study include the use of self-report and cardiovascular measures in a large sample of individuals in an esports performance context. Using this multi-method approach, we found that blunted cardiovascular reactivity may serve as an index of psychological task disengagement in motivated performance. Furthermore, we found that poor performances may increase psychological task engagement, and in turn, may increase physiological task engagement. These results broaden understanding of how task engagement and performance outcomes influence each other in multi-round performance.

## Data Availability

The datasets generated during and/or analyzed during the current study are available from the corresponding author on reasonable request.
